# Whole genome sequencing of *Enterobacter mori*, an emerging pathogen of kiwifruit and the potential genetic adaptation to pathogenic lifestyle

**DOI:** 10.1186/s13568-021-01290-w

**Published:** 2021-09-17

**Authors:** Mingyang Zhang, Yilin Zhang, Xue Han, Juan Wang, Yu Yang, Biao Ren, Mian Xia, Gang Li, Rongxiang Fang, Hang He, Yantao Jia

**Affiliations:** 1 State Key Laboratory of Plant Genomics, Institute of Microbiology, Chinese Academy of Sciences, Beijing, 100101 China; 2 National Plant Gene Research Center, Beijing, 100101 China; 3 School of Advanced Agriculture Sciences and School of Life Sciences, State Key Laboratory of Protein and Plant Gene Research, Peking University, Beijing, 100871 China; 4 College of Life Sciences, University of the Chinese Academy of Sciences, Beijing, 100049 China; 5 Cangxi Xingke Modern Agricultural Science and Technology Research Institute Co., Ltd., Cangxi, 628400 China; 6 Guangxi Key Laboratory of Medicinal Resource Protection and Genetic Improvement, Guangxi Botanical Garden of Medicinal Plant, Nanning, 530023 China

## Abstract

Members of the *Enterobacter* genus are gram-negative bacteria, which are used as plant growth-promoting bacteria, and increasingly recovered from economic plants as emerging pathogens. A new *Enterobacter mori* strain, designated CX01, was isolated as an emerging bacterial pathogen of a recent outbreak of kiwifruit canker-like disease in China. The main symptoms associated with this syndrome are bleeding cankers on the trunk and branch, and brown leaf spots. The genome sequence of *E*. *mori* CX01 was determined as a single chromosome of 4,966,908 bp with 4640 predicted open reading frames (ORFs). To better understand the features of the genus and its potential pathogenic mechanisms, five available *Enterobacter* genomes were compared and a pan-genome of 4870 COGs with 3158 core COGs were revealed. An important feature of the *E*. *mori* CX01 genome is that it lacks a type III secretion system often found in pathogenic bacteria, instead it is equipped with type I, II, and VI secretory systems. Besides, the genes encoding putative virulence effectors, two-component systems, nutrient acquisition systems, proteins involved in phytohormone synthesis, which may contribute to the virulence and adaption to the host plant niches are included. The genome sequence of *E*. *mori* CX01 has high similarity with that of *E. mori* LMG 25,706, though the rearrangements occur throughout two genomes. Further pathogenicity assay showed that both strains can either invade kiwifruit or mulberry, indicating they may have similar host range. Comparison with a closely related isolate enabled us to understand its pathogenesis and ecology.

**Supplementary Information:**

The online version contains supplementary material available at 10.1186/s13568-021-01290-w.

## Key points

A new bacterial pathogen (*Enterobacter* species) was isolated from diseased kiwifruit plant and characterized by 16S rDNA sequence.

The whole genome sequence of the isolate was composed of one circular chromosome. The genome comparison with other *Enterobacter *strains were performed.

The new bacterial causal agent *Enterobacter* *mori* CX01 may evolve new strategies to infect kiwifruit plants.

## Introduction

*Enterobacter* is a genus of gram-negative, rod-shaped and non-spore-forming bacteria belonging to the family *Enterobacteriaceae* (Hormaeche and Edwards 1960). *Enterobacter* bacteria are widely distributed in nature, some species function as plant growth promoting bacteria in agriculture (Majeed et al. 2018), while many of others are frequently isolated from hospitals and recognized as opportunistic pathogens causing various types of human infections (Mezzatesta et al. 2012). According to information in NCBI database, as of May 2021, 28 *Enterobacter* whole genome sequences were completed. Only two cases *E. mori* LMG 25706 and *E. ludwigii*, are phytopathogenic bacteria which cause diseases on mulberry and onion, respectively. The mulberry leaves infected by *E. mori* LMG 25706 became withered and dry, and then defoliated, the diseased root xylem was moist and discolored with brown stripes, the phloem decayed at the late stage of infection (Zhu et al. 2011). *Enterobacter* bulb decay caused by *E. ludwigii* (previous termed *E*. *cloacae*) is characterized by a light brown discoloration of the internal fleshy scales (Schroeder et al. 2010). It’s worth noting that *E*. *cloacae* and other species of this complex have been showing increasing importance as pathogens on various economic plants (e.g., papaya, cassava, rice and cotton) recently (Cao et al. 2020; Garcia-Gonzalez et al. 2018; Nagrale et al. 2020). However, no *Enterobacter* species associated with kiwifruit bacterial disease have been reported so far.

The kiwifruit (*Actinidia* spp.) is a worldwide economically and nutritionally important fruit crop. The kiwifruit industry has been seriously threatened by *Pseudomonas syringae* pv. *actinidiae* (*Psa*) (Donati et al. 2014). Canker disease caused by *Psa* was first identified and described in Japan in 1984 (Takikawa et al. 1989), and had also been reported to break out in 1989 in Cangxi, Sichuan, China (Wang et al. 1992). It is reported that many isolates of *P. syringae* that are phylogenetically closer related to *Psa* are nonpathogenic and exist as commensals on plants (Xin et al. 2018). The mechanism studies of *P. syringae* virulence factors and genomic and evolutionary insights have revealed basic features of *P. syringae* as a successful plant pathogen (Xin et al. 2018). Similarly, *Enterobacter* species commonly occur as pathogenic or nonpathogenic bacteria. Understanding the genetic basis of the pathogenic species by comparison with its closely related nonpathogenic bacteria, will help us elucidate the pathogenesis of these microorganisms.

In this study, we identified a new pathogen termed *E*. *mori* CX01 strain from diseased kiwifruit plant in Cangxi, Sichuan province of China. To our knowledge, this is the first report that *Enterobacter* species caused canker-like symptoms such as bleeding of kiwifruit vascular tissues and significant vine death. The whole genome sequence information of *E*. *mori* strain CX01 should provide the basis for a better understanding of its genetic background. Bioinformatic analysis results uncovered those diverse pathways may help *E*. *mori* perceive and respond to environmental cues, and survive in plant tissues.

## Materials and methods

### Bacterial collection and preliminary characterization

The symptomatic samples of kiwifruit trunks, branches and leaves were collected from five regions in Cangxi, Sichuan province, China. The pith juice was taken and diluted with 1 ml distilled water. Leaves were rinsed with sterile distilled water (SDW), disinfested with 0.3% sodium hypochlorite and rinsed with SDW four times. Small leaf sections were excised from the edge of the diseased tissue and macerated in SDW for 90 s in a sterile mortar. The above suspensions were streaked onto King’s B (KB) agar plates and incubated for 24 h at 28 °C (King et al. 1954). Then, a single colony was successively re-streaked onto a new KB agar plate to obtain a pure colony. Initially, the 16S rRNA gene was PCR-amplified with bacterial 16S rRNA universal primers 27F 5′-AGAGTTTGATCMTGGCTCAG-3′ and and 1492R 5′-GGTTACCTTGTTACGACTT-3′, 8F 5′-AGAGTTTGATCCTGGCTCAG-3′ and U1492R 5′-CGGTTACCTTGTTACGACTT-3′ (Edwards et al. 1989; Lane 1991) and 16S rRNA specific primer pairs of Em-16 s-F 5′-AGCACGTGTGTAGCCCTACTCGTA-3′ and Em-16 s-R 5′-AGAGATCTGGAGGAATACCGGTGGC-3′. The amplified PCR product was ligated into cloning vector pClone007 Simple (TSINGKE Biological Technology) and sequenced, then compared with reference sequences available in the GenBank/EMBL/DDBJ databases using BLAST to present the similarity with other bacterial species. Primer pairs of Em-16 s-F and Em-16 s-R were also used in amplicon-sequencing for detection *E*. *mori* in further collected samples. Primer pairs of Psa-16S-23F 5′-TTTTGCTTTGCACACCCGATTT-3′ and Psa-16S-23R 5′-CACGCACCCTTCAATCAGGATG-3′ were used to detect *Psa* strains.

### Pathogenicity tests

*E. mori* CX01 (CGMCC 1.19108) and LMG25706 (CGMCC 1.10322) inoculation experiments were conducted either on kiwifruit (cultivar Hayward) or on mulberry (variety Baisang) seedlings to confirm bacteria's pathogenicity. Kiwifruit and mulberry leaves were inoculated by scissor clipping with the bacterial suspensions (10^9^ colony-forming units/ml, cfu/ml), then transferred in a climatic chamber at 25 °C to detect the necrotic symptoms around the inoculated wounds 6–9 days after inoculation.

Kiwifruit aseptic seedlings were inoculated by vacuum infiltration with the bacterial suspensions (10^7^ cfu/ml), after treatment at 4–10 °C for 3–4 days, kiwifruit seedlings were transferred to 25 °C plant incubator. At 7–10 days after inoculation, the seedling leaves were ground up and spread on KB agar plates.

One- to two-year-old vines of kiwifruit (cv. Hongyang) were inoculated by puncturing methods. Fifteen vine sticks were pricked with a needle through a drop (200 μl) of bacterial suspension (10^9^ cfu/ml) placed on them, then incubated under 25 °C for 7 to 10 days to photograph the symptom.

In all above methods, sterile water-treated samples were taken as negative controls. Koch’s postulations were verified by characterizing *E*. *mori* re-isolated from the inoculated samples with similar symptoms. *E. mori* (CGMCC 1.10322) was bought from China General Microbiological Culture Collection Center.

### Genome sequencing and de novo assembly

Genomic DNA of *E. mori* CX01 (CGMCC 1.19108) was extracted from fresh bacterial cultures using a modified CTAB (cetyltrimethyl ammonium bromide) method (Murray and Thompson 1980). For single-molecule real-time (SMRT) sequencing (Pacific Biosciences), 15 µg DNA was fragmented by partial digestion with *Sau*3AI, and the DNA fragments at about 10–50 kb were selected for the library construction and further sequenced on the PacBio Sequel system (Pacific Biosciences, Menlo Park, CA, USA). At least 5 µg DNA was used to construct paired-end libraries (PE 150 bp) and sequenced on Illumina HISEQ platform. A total of ⁓ 1.86 Gb SMRT subreads and ⁓ 485 Mb PE reads were produced.

The genome size was estimated using PE reads by KmerGenie (v1.7048) (Chikhi and Medvedev 2014). PacBio subreads were assembled using Canu (v1.8) (Koren et al. 2017) and MECAT (v1.0) (Xiao et al. 2017) respectively, with default parameters and the estimated genome size of 4,961,053 obtained above. In order to compare two genome drafts, we used nucmer to generate alignments between two assemblies, and the draft assembled by MECAT has better continuity, therefore was selected for further analyses. In order to reduce the base error rate, we used the consensus algorithm Quiver and further polished the assembly with PE reads using Pilon.

### Gene prediction and functional annotation

The RAST server was used to annotate the *E*. *mori* CX01 draft genome (Darling et al. 2004). Genome annotation was carried out using the Rapid Annotations using Subsystems Technology (RAST) server with the RASTtk annotation scheme and default parameters (Brettin et al. 2015). The rRNAs of a new genome are simply found using a BLASTN (McGinnis and Madden 2004) search against this curated set which have phylogenetically diverse set of representative sequences of the 23S (currently 81 representatives), 16S (currently 120 representatives) and 5S (currently 292 representatives) rRNAs. RAST use the tRNAscan-SE (Lowe and Eddy 1997) to Calling tRNAs. The complete genome sequence of *E*. *mori* CX01 has been deposited to the NCBI database under the GenBank accession number CP055276. The KO numbers of the genes and the KEGG metabolic pathway for comparative genome was annotated by KAAS using blast program and database of Prokaryotes (Kanehisa and Goto 2000; Moriya et al. 2007).

### Phylogenomic analysis and genome comparison of* Enterobacter* strains

Genome alignment between *E*. *mori* CX01 and *E*. *mori* LMG 25706 was performed by using Mauve (Darling et al. 2004). In order to determine the evolutionary relationship of *E*. *mori* CX01 with other bacteria on the phylogenetic tree, four housekeeping genes (*gyrB*-*rpoB*-*atpd*-*infb*) of 44 strains from *Enterobacter* genus and other 9 different genera were selected to establish phylogenetic tree by neighbor-joining method using MEGA7 software (Kumar et al. 2016). The gene sequences of the selected bacteria are obtained from the NCBI database. The housekeeping gene sequences of *E*. *mori* CX01 were obtained by using known gene sequences as queries to blast their homologue genes in the genome with Blastn (McGinnis and Madden 2004).

Gene family clustering was performed using Orthovenn2 under default parameters (Xu et al. 2019) on protein sequences of *Enterobacter mori* CX01, *Enterobacter mori* LMG 25706, *Enterobacter cloacae* (NC_014121.1), *Enterobacter cancerogenus* (NZ_CP025225.1) and *Enterobacter hormaechei* (NZ_CP017179.1). The Orthovenn2 software also provides GO enrichment analysis for specific clusters (Xu et al. 2019).

## Results

### Isolation and characterization of a pathogenic bacterium from kiwifruit plants

**Fig. 1:**
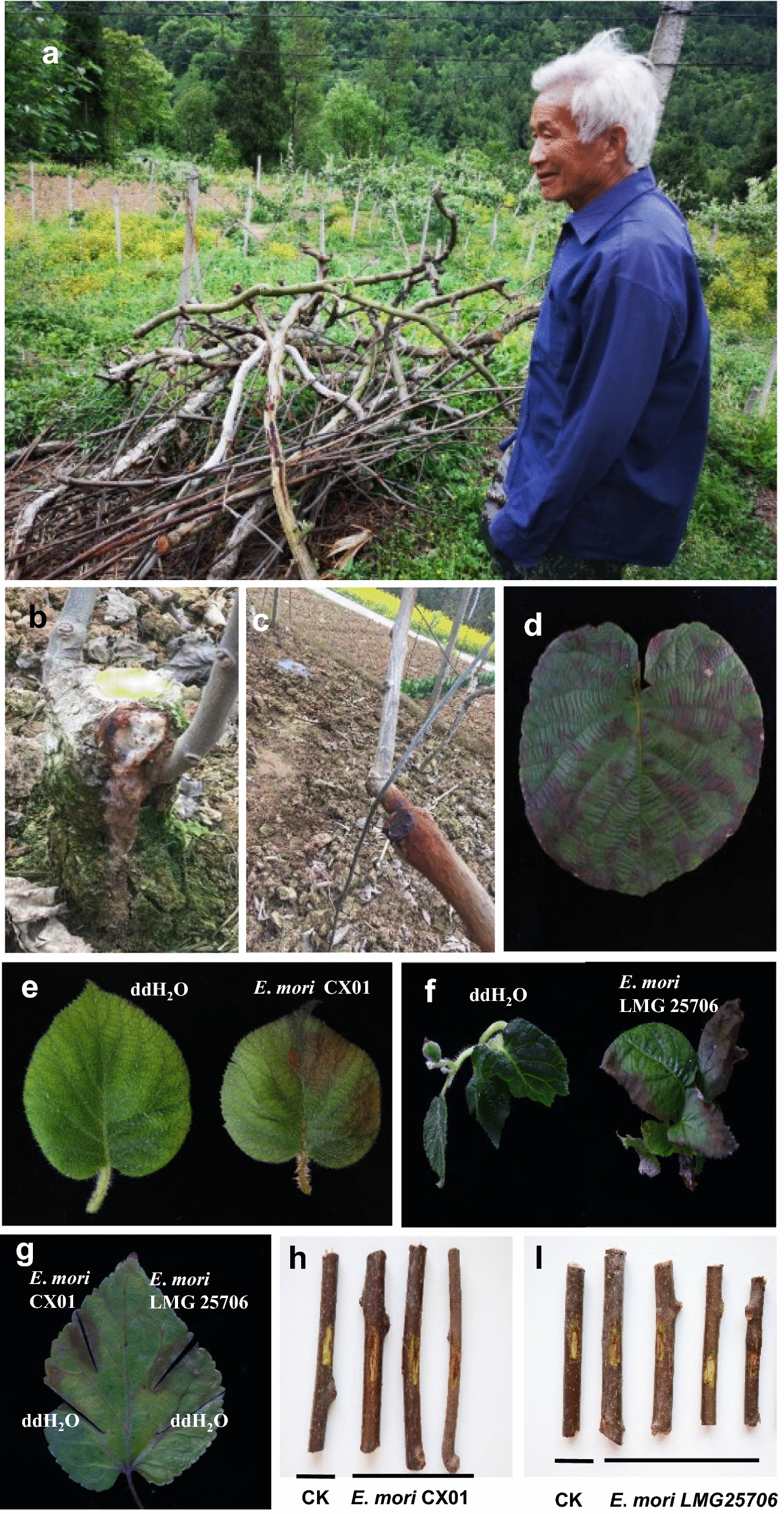
Disease symptom of *E*. *mori* on plants and its epidemics in kiwifruit orchards of Cangxi. **a** High disease incidence caused by *E*. *mori* led to eradication of whole kiwifruit orchards. **b** The canker-like disease symptoms on the trunk with white exudation. **c** Reddish-brown discoloration under the bark of the trunk. **d** Disease symptom of brown spots on kiwifruit leaf. Disease symptom of kiwifruit (*Actinidia deliciosa* cv. Hayward) leaf after inoculation of *E*. *mori* CX01 (**e**) or *E*. *mori* LMG25706 (**f**). **g** The symptoms of *E*. *mori* CX01 and LMG25706 inoculated mulberry (*Morus alba* L.)leaf. **h** and **I** Twigs (*Actinidia chinensis* cv. Hongyang) showing brownish necrosis after puncturing inoculation with *E*. *mori* CX01 or LMG25706

In 2018, a serious bacterial disease was investigated at 50 naturally infected kiwifruit orchards in Cangxi, Sichuan Province, China. The disease incidence in the field of various cultivars ranged from 50 to 80%, the highest of which was associated with the *Actinidia chinensis* cultivars, in particular with red-fleshed kiwifruit cultivar ‘Hongyang’ (always up to 80%). Wounds and freezing led to more serious symptoms, and eradication of the whole orchard had to be carried out due to the extremely high percentage of disease (Fig. 1a). The main symptoms of the disease were white or brown fluid bleeding cankers in the bark on the trunk and branch during early spring (then termed canker-like disease), brown leaf spots also appeared in summer (Fig. 1b–d), which is similar to the seasonal occurrence rhythm and phenomena of the canker disease caused by *Psa* (Donati et al. 2014). In severe infections, twig wilting and plant death were observed.

More than 100 bacterial isolates were obtained from 45 diseased trunks and 60 leaves of 1–5-year-old kiwifruit plants, and at least 50 recovered cultures collected from 15 different plants in 5 orchards were analyzed. Surprisingly, no *Psa* strain was found in these collected tissues. The causal agent of the canker-like disease was identified to belong to the genus *Enterobacter* based on 16S ribosomal RNA (rRNA) gene sequencing performed by using 16S rDNA universal primers (27F/1492R and 8F/U1492R), and further confirmed the molecular characterization of 20 randomly chosen isolates obtained from 8 different plants of 5 orchards by *Enterobacter* 16S rDNA primer pairs of Em-16s-F and Em-16s-R. The results showed that the bacterial 16S rDNA sequences have the highest identity of 99.2% with that of *E*. *mori* LMG 25706 which causes wilt disease on mulberry (Zhu et al. 2011). According to the amended criteria for bacterial species classification, the homology of 16S rDNA sequences reaches 98.7–99% could be classified into the same species (Stackebrandt and Ebers 2006). Then, the new pathogenic bacterium strain was named as *E*. *mori* CX01. *E*. *mori* is gram-negative, facultatively anaerobic, rod-shaped, and motile with polar flagella.

To detect the pathogenicity of *E*. *mori* CX01 and *E*. *mori* LMG 25706 on kiwifruit, healthy kiwifruit seedlings were inoculated by vacuum infiltration with the suspension of bacterial cells. At least 30 leaves showed brown necrotic phenotype after 7 days of infection by *E*. *mori* CX01 or *E*. *mori* LMG 25706 (Fig. 1e, f). Both *E*. *mori* strains could be recovered from the infected leaves. We found that mulberry plants are also susceptible hosts of *E*. *mori* CX01 as evaluated by leaf clipping inoculation. At least 10 mulberry leaves were scissor-clipped with bacterial cells of the two strains, dark-brown edges on both sides of wounding leaves were shown after 7 days, whereas water-treatment control remains healthy (Fig. 1g). Since the main symptoms caused by *E*. *mori* CX01 was found in vascular tissues, *E*. *mori* CX01 and LMG 25706 were also puncture-inoculated on 15 kiwifruit healthy twigs. Brown lesions were observed at the inoculated sites after 7 days (Fig. 1h, I). The re-isolated bacteria from the diseased tissues were confirmed by 16S rRNA gene sequencing. These results fulfilled the Koch’s postulates.

### Whole-genome sequencing and phylogenetic analysis of *E*. *mori*

The genomic DNA of *E*. *mori* CX01 was subjected to whole-genome sequencing, which resulted in 1,46,372 high-quality filtered reads of an average read length of 12,681 bp and coverage equivalent to about 372 times. Quality filtered reads were assembled into scaffolds. The complete genome sequence of *E*. *mori* CX01 has been deposited to the NCBI database under the GenBank accession number CP055276. The *E. mori* CX01 genome has a 4,966,908 base-pair circular chromosome with a 55.40% G + C content. A total of 4640 open reading frames (ORFs) were predicted and annotated. A large proportion of ORFs have been annotated GO (gene ontology) terms. The chromosome has eight 16S rRNA genes and 84 tRNAs genes, and 4282 ORFs (92.28%). The genome reveals the existence of two putative CRISPR (Clustered Regularly Interspaced Short Palindromic Repeat) arrays with the highest evidence level of 4, and no Cas protein detected in the genome by CRISPRCasFinder prediction (Additional file 1: Figure S1).

**Fig. 2:**
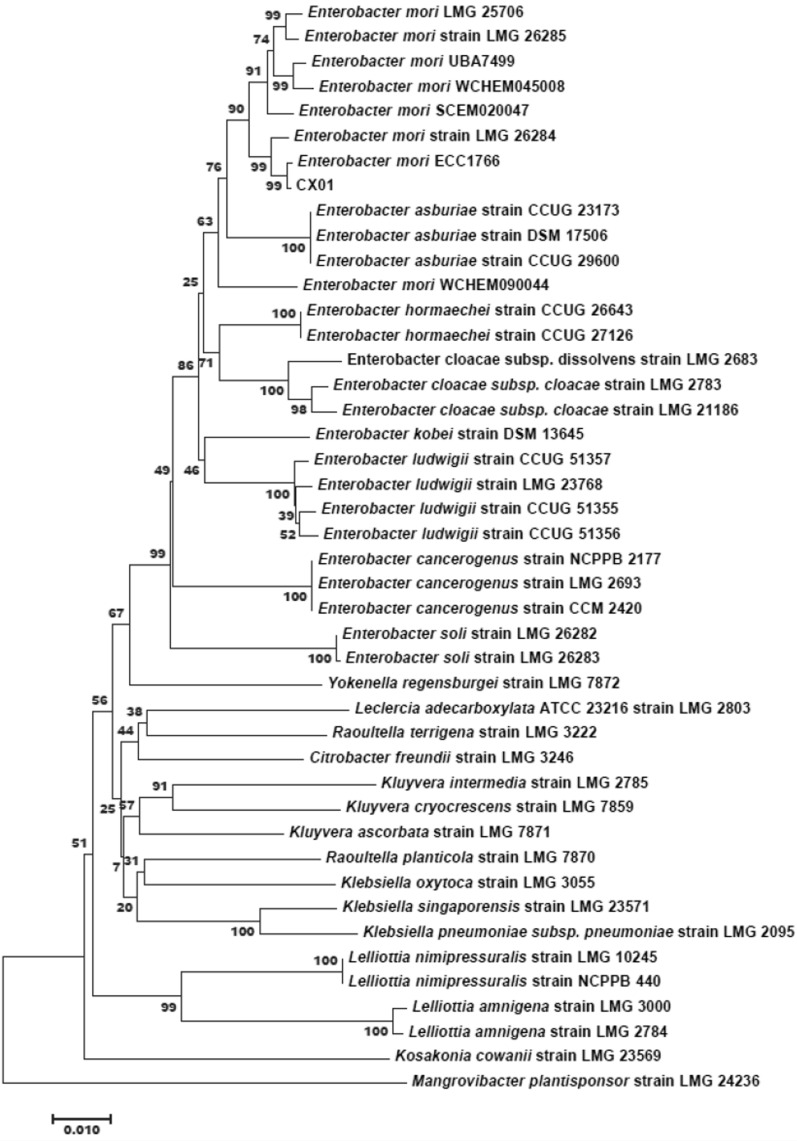
Phylogenomic analysis of *Enterobacter* and other genus bacteria. Phylogenetic tree reconstructed from multilocus sequence analysis (MLSA) based on four housekeeping genes *gyrB*-*rpoB*-*atpD*-*infB*, showing the relationships between *E*. *mori* CX01 and the related type strains. The phylogenetic tree was inferred by the neighbor-joining method with the software MEGA7 (Kumar et al. 2016). The bar indicates a 0.01% nucleotide substitution rate

Phylogenetic analysis was performed by using concatenated nucleotide sequences of housekeeping genes (*infB*, *gyrB*, *atpD* and *rpoB*) (Fig. 2, Additional file 2: Table S1). MLSA (multilocus sequence analysis) analysis showed that different *Enterobacter* species clustered together on the phylogenetic tree. The subgroup of *E*. *mori* species includes *E*. *mori* CX01 and seven other strains (the phytopathogens of *E*. *mori* LMG 25706, LMG 26284, LMG 26285 and UBA7499, three opportunistic human pathogens of SCEM020047, WCHEM045008 and ECC 1766). The subgroup of *E*. *asburiae* represented by three clinical strains, which is closely related to *E*. *mori* subgroup. Other *Enterobacter* species such as *E*. *cloacae*, *E*. *hormaechei*, *E*. *cancerogenus* and *E*. *ludwigii*, most of them were collected from hospital samples, *E*. *cloacae* subsp. *dissolvens* LMG 2683, *E*. *cancerogenus* and *E*. *soli* were isolated from *Zea mays*, *Populus* and *Eucalyptus*, respectively.

**Fig. 3:**
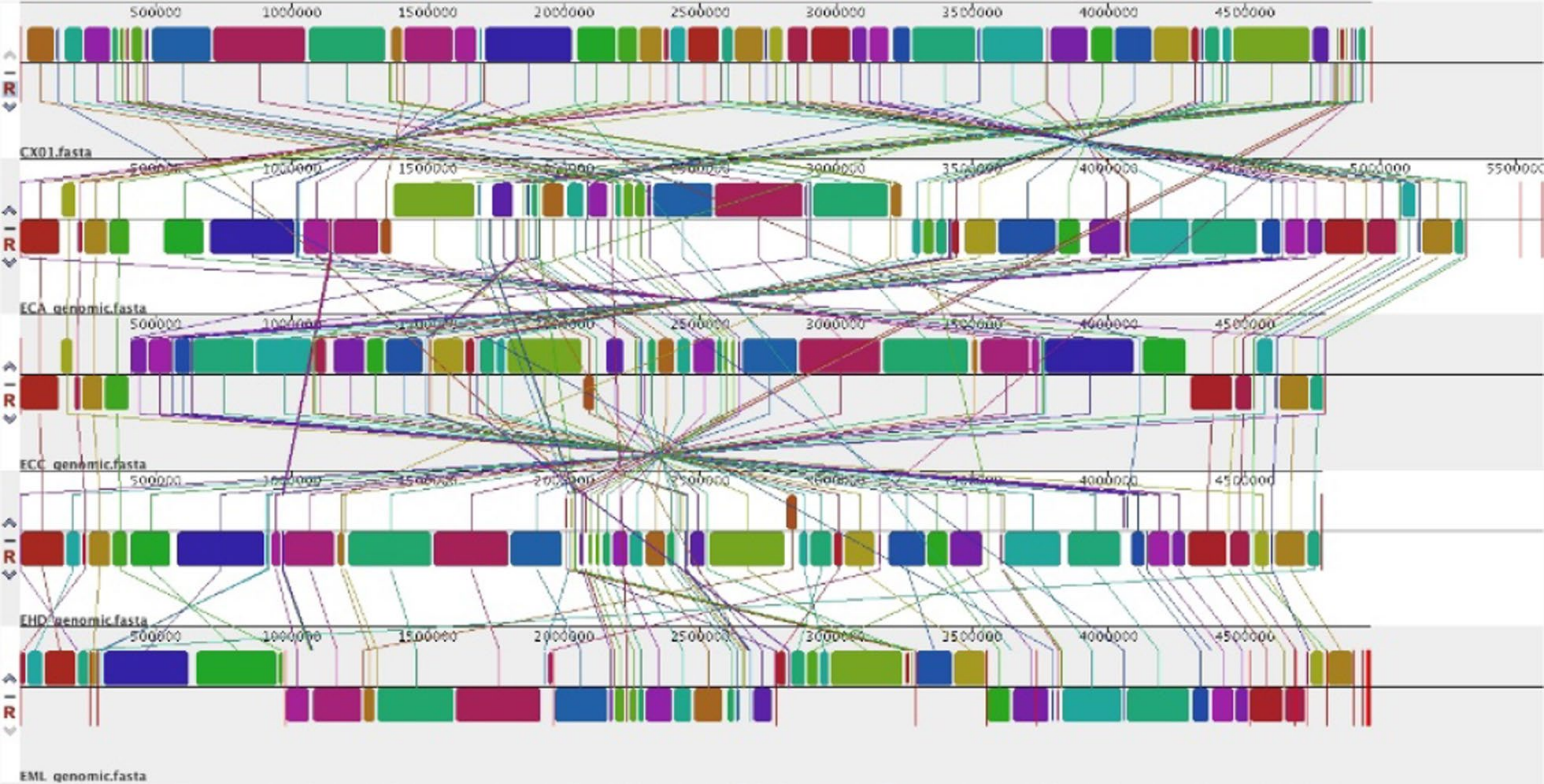
Genomic alignment of *Enterobacter* species. Alignment of the genome sequences of *E. mori* LMG 25706 (EML), *E. mori* CX01 (CX01, CP055276), *E. cloacae* ATCC 13047 (ECA, NC_014121.1), *E. hormaechei* DSM 16691 (EHD, NZ_CP017179.1) and *E. cancerogenus* CR-Eb1 (ECC, NZ_CP025225.1) with MAUVE software (Darling et al. 2004). Same color boxes represent homologous regions of the sequences, and are connected by lines

**Fig. 4:**
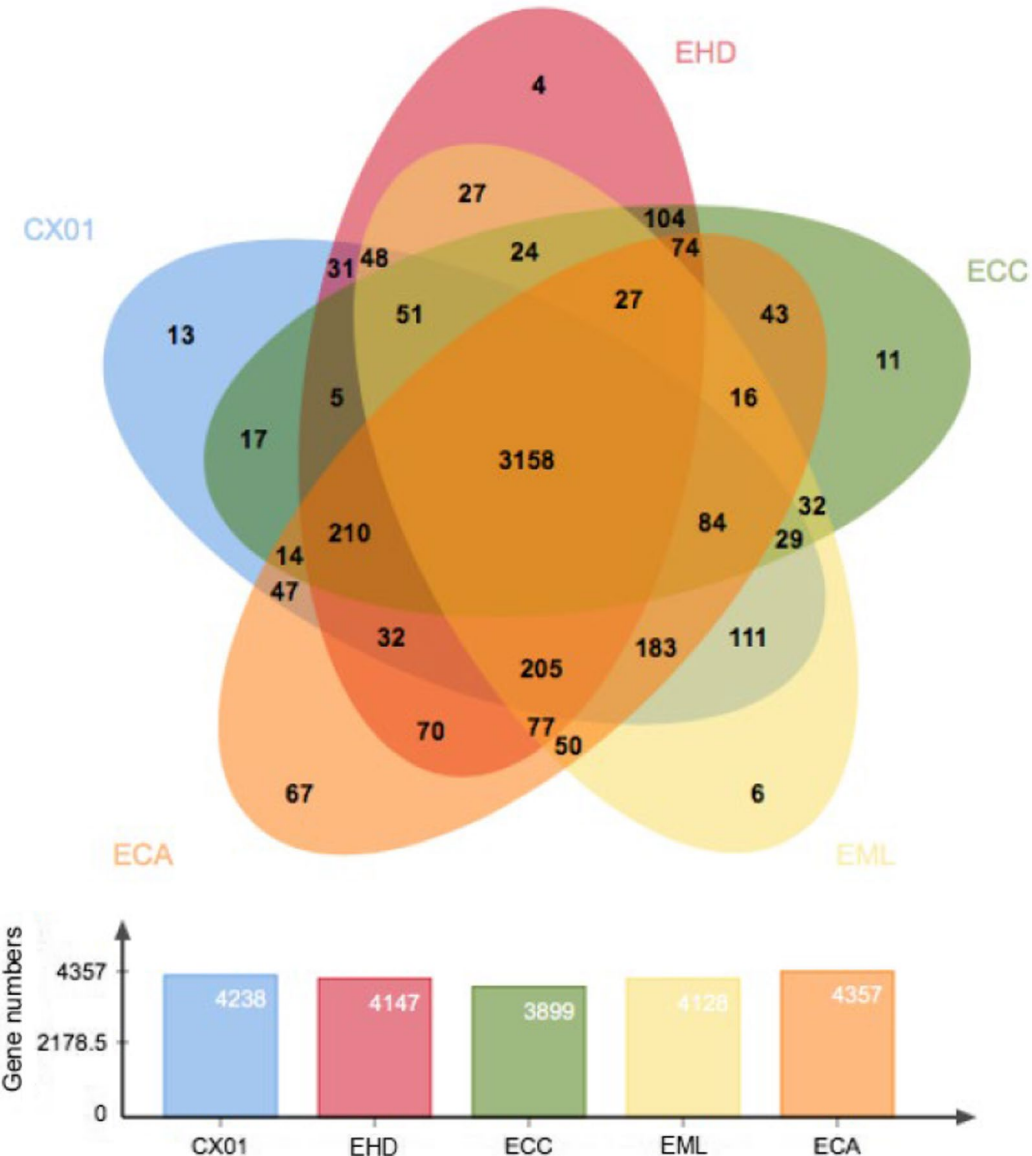
Comparison of COGs in five *Enterobacter* species. The numbers in overlapped regions of Venn diagram represent CDS numbers shared by the genomes, including *E. mori* LMG 25706 (EML), *E. mori* CX01 (CX01, CP055276), *E. cloacae* ATCC 13047 (ECA, NC_014121.1), *E. hormaechei* DSM 16691 (EHD, NZ_CP017179.1) and *E. cancerogenus* CR-Eb1 (ECC, NZ_CP025225.1)

We further performed genome alignment to investigate a collinear relationship among five *Enterobacter* strains by MAUVE analysis (Fig. 3). Genomic comparison of two phytopathogenic strains (*E*. *mori* CX01 and *E*. *mori* LMG 25,706) with three clinical isolates, *E*. *cloacae* (accession number NC_014121.1), *E*. *cancerogenus* (accession number NZ_CP025225.1) and *E*. *hormaechei* (accession number NZ_CP017179.1), revealed the presence of a large core-genome of 3158 COGs (cluster of orthologous groups), and the pan-genome of 4870 COGs as shown in Fig. 4. A set of 111 COGs specifically belongs to *E. mori* CX01 and LMG 25706, involved in multiple metabolic pathways, including gene expression regulation, biosynthetic and catabolic processes, substrate transport, response to biotic and abiotic stresses and cytolysis process (Additional file 2: Table S2). In *E. mori* CX01, 224 coding sequences (CDSs) included in the 111 COG classes that arranged in seven fragments, the average GC content of two fragments is 45.7% and 39.7%, which is much lower than that of the entire genome (55.40%), and most likely concerned with horizontal gene transfer events. One fragment (EM_0117-0135) encodes for AraC family transcriptional regulator, dienelactone hydrolase, superfamily I DNA and RNA helicase, phage integrase, DNA-binding protein, cold shock protein, glucan synthase, Lipid A biosynthesis palmitoleoyltransferase and hypothetical proteins. The other fragment (EM_1323-1329) encodes Rhs-family protein, two tetrapartite efflux system proteins, AraC family transcriptional regulator and hypothetical proteins. In addition, 13 COGs (including 33 CDSs) from *E. mori* CX01 were classified as unique, besides hypothetical proteins, 6 GO (gene ontology) terms were represented involved in transposition, hydroxypyruvate reductase activity, isomerase activity, transmembrane transport, phosphoenolpyruvate-dependent sugar phosphotransferase system and sequence-specific DNA binding (Additional file 2: Table S3). While 6 COGs (including 12 CDSs) were unique to the LMG 25,706 genome (Additional file 2: Table S4) involved in respiratory electron transport chain, protein oxidation, virus tail/fiber and transmembrane transport.

**Fig. 5:**
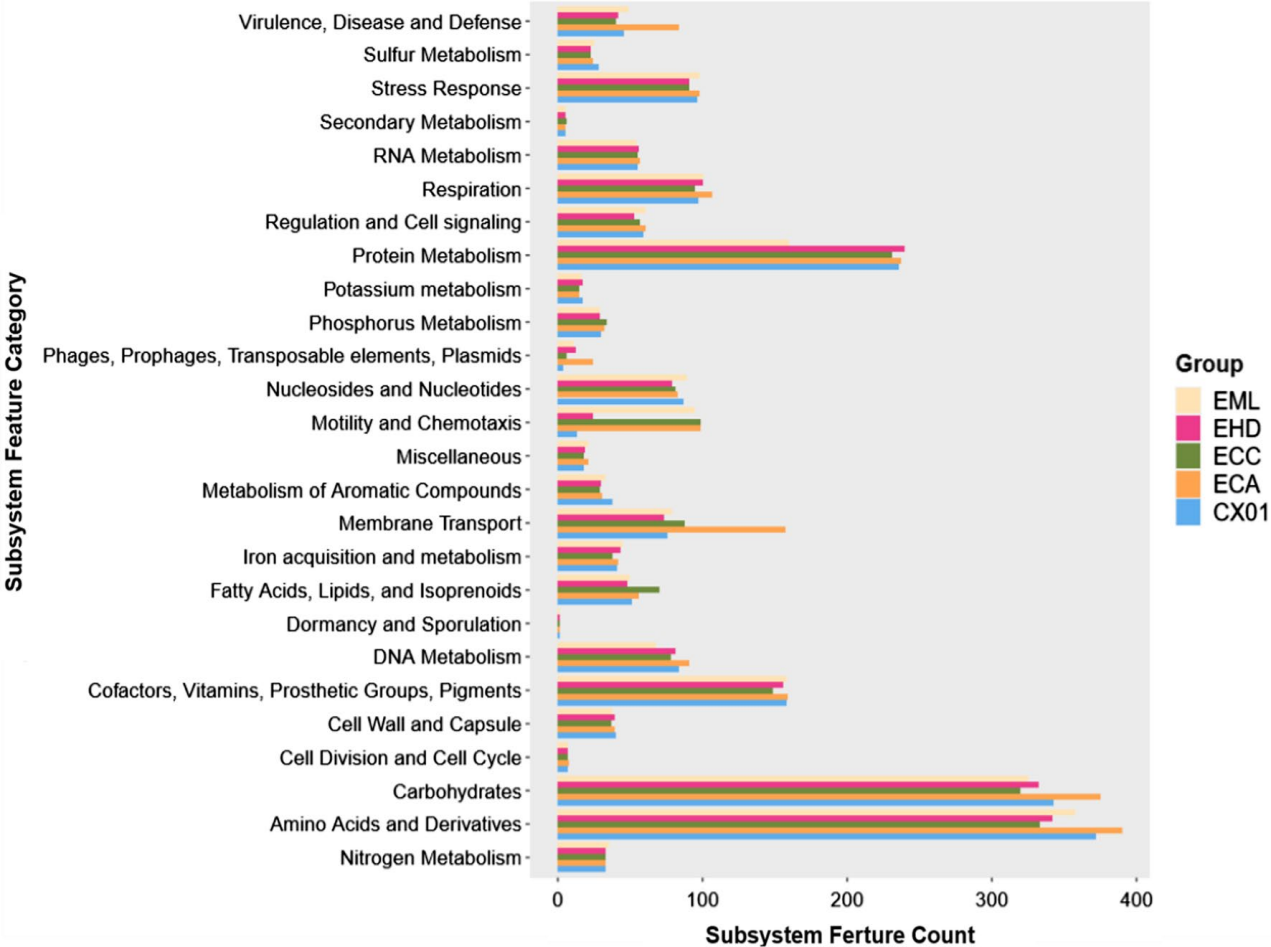
Comparison of subsystem features among *Enterobacter* species. The data are derived from genome information of *E. mori* LMG 25706 (EML), *E. mori* CX01 (CX01, CP055276), *E. cloacae* ATCC 13047 (ECA, NC_014121.1), *E. hormaechei* DSM 16691 (EHD, NZ_CP017179.1) and *E. cancerogenus* CR-Eb1 (ECC, NZ_CP025225.1)

Metabolic pathways shown in subsystem feature categories are conserved among these five *Enterobacter* species that are characterized by the capability of using diverse carbon sources through multiple carbohydrate metabolic pathways. Over 320 genes were assigned for the role of carbohydrate utilization, accounting for about 7% of the genes in *E*. *mori* CX01 and LMG 25706 (Fig. 5) (Additional file 2: Table S5), and more than 510 genes were significantly enriched in protein metabolism, amino acids and derivatives pathways accounting for 11–13% of the gene numbers of the two genomes (Additional file 2: Table S6).

### Genomic analysis reveals that *E. mori* CX01 is likely to be better equipped for growth in kiwifruit

Type III secretion system (T3SS) and Type IV secretion system (T4SS) were absent in *E*. *mori* CX01. Other secretion systems including type I, II, and VI secretion systems appeared in the genome, which are believed to be also related to the bacterial virulence (Green and Mecsas 2016). *E*. *mori* CX01 has integrate type I secretion system (T1SS) which is conserved in plant and animal bacterial pathogens. Small molecules such as antibiotics and toxins, proteases and lipases, adhesins, and proteins with repeats-in-toxins (RTX) motifs can be exported by T1SS (Green and Mecsas 2016). Type II secretion system (T2SS) that transports folded proteins from the periplasm into the extracellular environment, is important for both pathogenic and non-pathogenic bacteria (Green and Mecsas 2016). In some bacteria, T2SS is still functional in absence of important T2SS genes (de Vrind et al. 2003). Similarly, *E*. *mori* CX01 has incomplete T2SS core genes (*gspLKJHGFDE*), whether *E*. *mori* CX01 has a functional T2SS remains elusive. Type VI secretion system (T6SS) commonly translocates proteins into other bacteria cells, as well as eukaryotic cell targets (Green and Mecsas 2016). T6SSs are hypothesized to contribute to the virulence of some bacterial pathogens, by delivering protein effectors into host cells, and secreting substrates into neighboring bacteria that benefit for interbacterial competing in a specific host niche (Green and Mecsas 2016). The homologues of Hcp (hemolysin coregulated protein) and VgrG (valine glycine repeat) are present in *E*. *mori* CX01, which are secreted proteins of T6SS (Zheng and Leung 2007). Flagella play critical roles in infection processes such as motility which allows bacterium to move through the soil matrix and inside the plant, as well as in adhesion, biofilm formation, secretion of effector molecules, penetration through tissue structures to entry into eukaryotic cells (Chaban et al. 2015). We found that *E. mori* CX01 codes for flagellar genes (Additional file 2: Table S7), which may contribute to the bacterial pathogenesis. The Sec and Tat secretion pathways are highly conserved in bacteria, which were also identified in *E. mori* CX01.

As a phytopathogen, *E. mori* CX01 genome codes for virulence effectors, such as *srfBC* (EM_4592-4593), which are homolog proteins of *Salmonella enterica* involved in host colonization (Worley et al. 2000). Other putative virulence factors include VirK (EM_0966, EM_4235) whose homologue in *Agrobaterium tumefaciens* is associated with type IV secretion (Kalogeraki and Winans 1998), and MviM (EM_0755) which is putative oxidoreductase, and a predicted virulence factor (EM_4594) which is a homologue of SsrAB-activated protein. Pectin is a structural polysaccharide that is integral for the stability of plant cell walls. Pectinesterase (EM_1118) and pectin degradation protein (EM_3329) were identified in *E*. *mori* CX01.

Two-component systems (TCSs) mediate environmental signals to cellular responses signaling transduction commonly found in bacteria, archaea and a few eukaryotes (Stock et al. 2000). In *E*. *mori* CX01, 26 HKs (3 of which are hybrid kinases) and 31 RRs were identified (Additional file 2: Table S8). The homologs of YpdA/YpdB system in *Escherichia coli* contributes to nutrient scavenging before entry into stationary phase (Fried et al. 2013). QseB/QseC system is conserved in many bacterial species, and functions as a global regulator of virulence associated with quorum-sensing signaling cascade (Weigel and Demuth 2016). GlrR/GlrK up-regulates transcription of the *glmY/glmZ* sRNA to maintain normal synthesis of the cell wall and outer membrane when cells enter the stationary growth phase (Goepel et al. 2011). CitA/CitB system may modulate the expression of the downstream gene cluster (*citCDEFXG* of CX01) in response to citrate, and CitA/CitB presents in one regulatory cascade with DcuS/DcuR system which induces the expression of genes for fumarate respiration in *E*. *coli* (Scheu et al. 2012). In addition, a high number of TCSs have been evolved and integrated into diverse cellular signaling circuits, such as nutrient up-taking, response to metal ions or osmotic stress, which confer the bacteria with different traits that enhance their fitness for invading host plants.

Nutrient acquisition is important for bacterial habitation in plant tissues. Consistently, a large number of transporters are present in *E*. *mori* CX01 that may allow the exchange of various substrates. There are more than 200 genes coding for ABC transporters that may be involved in the uptake of metals, osmoprotectant, sugar, amino acids, peptides, polyamines, vitamins, phosphate, sulphate, nitrate/nitrite, urea and quorum sensing autoinducer-2 (AI-2) in *E*. *mori* CX01 (Additional file 2: Table S9). *E*. *mori* CX01 also has the potential to incorporate a wide range of sugars through PTS systems (encoded by 53 genes) and MFS transporters (encoded by 59 genes) (Additional file 2: Tables S10, 11). In addition, bacteria have evolved strategies to compete for iron that is required for bacterial growth and full virulence of pathogens (Payne 1988). The iron uptake system includes specific iron uptake transporters, siderophores for iron chelating and siderophore receptors for utilizing heterologous siderophores. *E*. *mori* CX01 contains two ferrous iron uptake transporters, FeoABC (EM_2744–2746) and EfeBUO (EM_0794–0796), one ferrous-ion efflux pump FieF (EM_2504), two afuABC ferric-iron ABC transporters (EM_1356–1358, EM_1528–1530), fepABCDEG ferric enterobactin transporter (EM_1269, EM_1261, EM_1265, EM_1263, EM_3328, EM_1264), fhuBCD ferric hydroxamate transporter (EM_1655–1657). SitABCD (EM_1271–1274) and MntH (EM_3750) are manganese importer, and ZupT (EM_3116) zinc transporter. Ferritin family of iron storage proteins are present in all types of cells from higher eukaryotes to bacteria for dealing with the dual problem of iron availability and toxicity (Bradley et al. 2014). *E*. *mori* CX01 encodes bacterioferrin (EM_2810) and two ferrin-like protein (EM_4163, EM_4168) as iron-storage proteins. TonB-dependent siderophore receptor (EM_4081) is also present in the genome. Siderophore receptors are important components for bacterial virulence (Payne 1988), the homologue protein ferric-enterobactin (iron-bound siderophore) of *E*. *mori* (EM_1269) is associated with siderophore receptor in *Enterobacter*.

Many plant-associated bacteria affect plant growth by the production of phytohormones. For example, bacteria enhance plant growth by the synthesis of the plant auxin indole acetic acid (IAA), from tryptophan via three alternative pathways (Taghavi et al. 2009). *E*. *mori* CX01 encodes indolpyruvate decarboxylase IpdC (EM_3753) and the amine oxidase (EM_0329), which may be involved in two IAA synthesis pathways and the intermediate molecules may be the source for IAA biosynthesis in plant. The acetolactate decarboxylase (EM_1299) and 2,3-butanediol dehydrogenase (EM_1297) may be responsible for catalyzing the production of acetoin and 2,3-butanediol. *E*. *mori* CX01 contains two isochorismate synthase (*ICS*) homolog genes (EM_3834 and EM_1206), which are key enzymes for synthesis of salicylic acid (SA). Polyamines are widely distributed in bacteria, plants and animals, and play important roles in cell proliferation, tissue growth and differentiation (Tabor and Tabor 1984). Accordingly, in the genome of *E*. *mori* CX01, there are genes coding for arginine decarboxylase (SpeA, EM_3221), agmatinase (SpeB, EM_3222) ornithine decarboxylase (SpeC, EM_1172, EM_3187), S-adenosylmethionine decarboxylase (SpeD, EM_1685) and spermidine synthase (SpeE, EM_1684), which may allow the synthesis of polyamines of putrescine and spermidine.

In *E*. *mori* CX01, a variety of enzymes and regulators help bacteria to survive upon oxidative stress, including three superoxide dismutases (EM_0495, EM_0506, EM_2499), two catalases KatE (EM_0597) and KatG (EM_2529), four hydroperoxide reductases (EM_1232, EM_1233, EM_1486, EM_1836), two non-heme chloroperoxidases (EM_866, EM_1898), two thiol peroxidases (EM_3702, EM_4473), two glutathione peroxidases (EM_0311, EM_0577), glutathione reductase (EM_2640), five glutathione S-transferases (EM_0385, EM_0482, EM_1035, EM_2125, EM_3798), and three γ-glutamyl transpeptidase (EM_0837, EM_1565, EM_2716).

### Potential nutrient depletion ability between *E. mori* CX01 and *Psa*

**Fig. 6:**
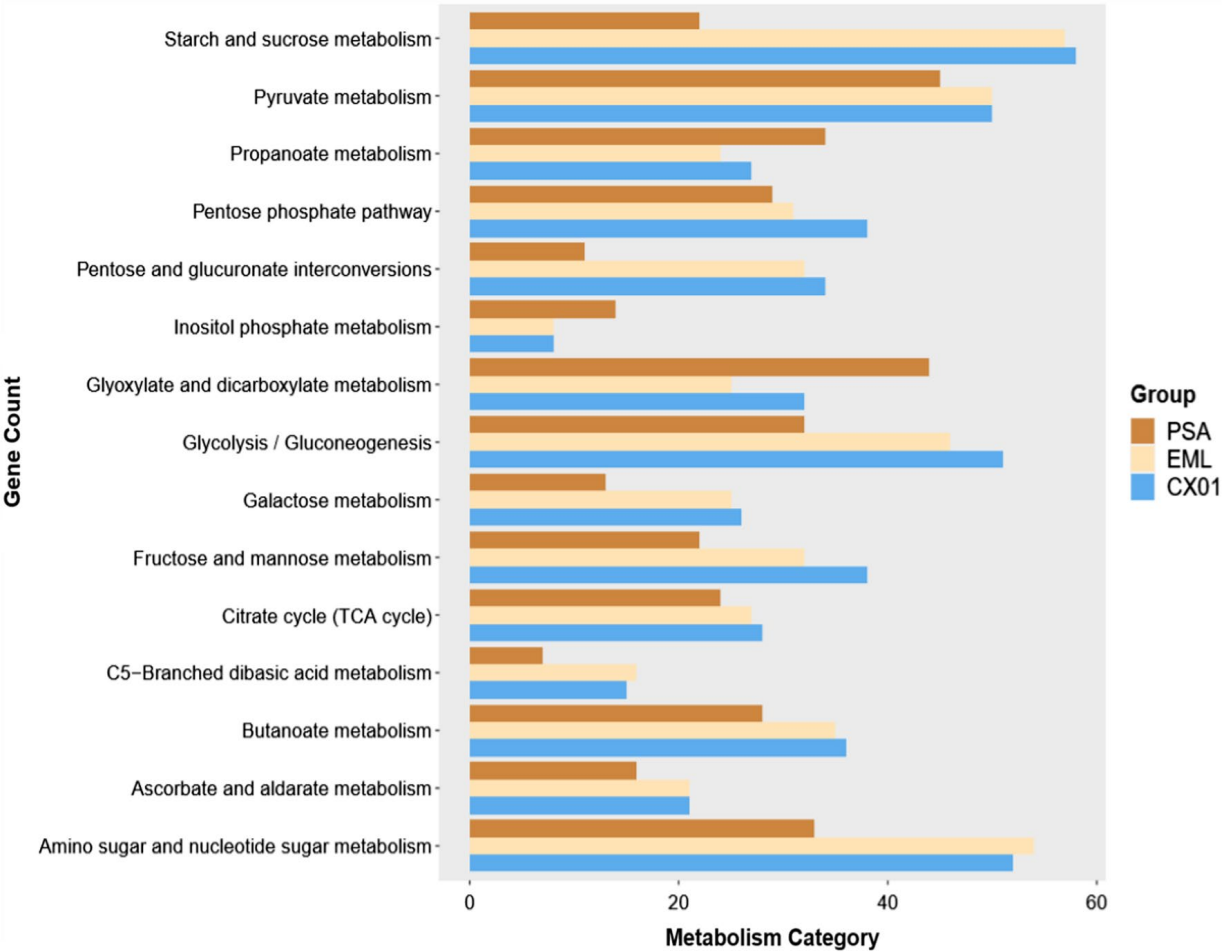
Functional KEGG metabolism pathway classification of predicted genes. The columns represent gene counts in *E. mori* LMG 25706 (EML)*, E. mori* CX01 (CX01) and *Psa* (PSA)

Nutrient depletion is a mode of bacterial competition, *E*. *mori* CX01 may have the potential of metabolizing a wide range of carbon substrates based on KEGG analysis, and the coding gene number is greater than that of *Psa* (Fig. 6). A total of 153 proteins was present in *E*. *mori* CX01, which are involved in pentose and glucuronate interconversions, fructose and mannose metabolism, galactose metabolism, starch and sucrose metabolism, amino sugar and nucleotide sugar metabolism, and C5-branched dibasic acid metabolism (Additional file 2: Table S12). The analysis also revealed 48 additional genes of the phosphotransferase system (PTS) specifically enriched in *E*. *mori* CX01 compared to *Psa* (Additional file 2: Table S13). *E*. *mori* CX01 encodes 30 additional biofilm formation genes compared with *Psa* (Additional file 2: Table S14). These genes include six cyclic diguanosine monophosphate (c-di-GMP) metabolism genes, four flagellar-related genes, seven polysaccharide biosynthesis/export protein genes, one operon for curli production, eight two-component system genes and transcriptional regulator genes. In the peptidase and inhibitor activity category, 90 genes of *E*. *mori* CX01 and 58 genes of *Psa* were classified into this KEGG pathway. *E*. *mori* CX01 contains a more diverse set of proteases compared with *Psa*, including out membrane protease, endopeptidase, L,D-transpeptidase, dipeptidase, aminopeptidase, and metalloprotease (Additional file 2: Table S15). An unique gene in *E*. *mori* CX01 encodes leader peptidase HopD (prepilin peptidase) (EM_2811). HopD homolog in *Legionella pneumophila* can cleave the leader sequences from prepilin-like proteins that are required for type II protein secretion, and are indispensable for the virulence of the pathogen (Liles et al. 1999).

*E*. *mori* CX01 and *Psa* possess 635 and 546 transporter genes respectively. The extremely high number of transporter genes in *E*. *mori* CX01 indicates it may translocate more diverse types of substrates than *Psa*. The additional transporters carried by *E*. *mori* CX01 include ATP-binding cassette subfamily B and C proteins, PTS and MFS transporters, as well as transport systems for nitrate/nitrite, thiamine, spermidine/putrescine, maltose/maltodextrin, arabinogalactan oligomer/maltooligosaccharide, maltose/maltodextrin, iron, manganese, amino acids and peptides (Additional file 2: Table S16). *E*. *mori* CX01 may utilize a variety of transport machines to easily access nutrient and survive in different environments.

The number of transcriptional regulators in *E*. *mori* CX01 (151) is higher than that of *Psa* (103), respectively. The additional members of transcriptional regulator in *E*. *mori* CX01 include AraC, LysR, LacI, GntR, TetR/AcrR, LuxR, MarR, MerR, IclR, Rrf2, XRE, DtxR, TrpR, SgrR, and FrmR/RcnR family transcriptional regulators (activator or repressor) (Additional file 2: Table S17), which may contribute to substrates utilization, stress responses, and multidrug efflux system, etc.

## Discussion

In this study, a new bacteria pathogen *E*. *mori* led to kiwifruit bacterial disease in Cangxi, Sichuan province of China. The whole genome of the isolate *E*. *mori* CX01 reveals its adaptative features to survive in plant niches (Additional file 3 and 4). *E*. *mori* CX01 as a pathogen to achieve the goal of host invasion, needs to initially disarm plant basal defense networks and subsequently to liberate nutrients required for its own multiplication. We found that is represented by the metabolic potential of *E*. *mori* CX01, such as secretion systems, putative virulence effectors, two-component systems, nutrient uptake systems, phytohormone synthesis and repression of host immunity.

*E*. *mori* CX01 lacks T3SS that is considered as a prerequisite for bacterial active virulent life style in many plant pathogens (Buettner and He 2009). *E*. *mori* can easily enter into the internal tissues of its host through wounds, so T3SS may not be nessesary for its invasion. Other secretion systems including type I, II, VI secretion system, which are believed to be also related to the bacterial virulence (Green and Mecsas 2016), are coded by *E*. *mori* CX01. Besides that, the flagellar protein export apparatus are harbored by *E*. *mori* CX01, which may also exert the function of secretion of virulence factors as many different pathogens, such as *Salmonella*, *Shigella*, *Serratia*, and *Yersinia* (Macnab 2003; Soutourina and Bertin 2003; Young et al. 1999). We noted that several putative virulence factors such as *srfBC*, VirK, MviM and pectinesterase were predicted in *E*. *mori* CX01, which may be secreted by above mentioned secretion systems and involve in the bacterial virulence.

In addition, bacterial pathogens need quickly adapt to changing environments during host entry or in a harsh environmental condition. This may be achieved by multiple signal response systems, such as two-component systems, to govern correct spatiotemporal gene expression in in *E*. *mori* CX01. A large number of two-component system genes are highly conserved within the genus *Enterobacter* (Capra and Laub 2012), indicating that the important role of two-component system in *Enterobacter* species. *E*. *mori* CX01 has the potential of producing volatile organic compounds acetoin and 2,3-butanediol (by acetolactate decarboxylase and 2,3-butanediol dehydrogenase), which are known to be required for full virulence in *Pectobacterium carotovorum* and increase the production of phenazine pyocyanin in *Pseudomonas aeruginosa* (Audrain et al. 2015). By contrast, some bacterial strains of plant growth-promoting rhizobacteria emitted 2,3-butanediol to induce systemic resistance in *Arabidopsis thaliana* (Ryu et al. 2004). Sucrose as the abundant nutrient can induce acetoin and 2,3-butanediol production in *Enterobacter* sp. 638 (Taghavi et al. 2009). In plant–microbe recognition, the ability of scavenging iron is also required for bacterial progression of disease symptoms on the host plants (Franza and Expert 2013). *E*. *mori* CX01 possesses iron uptake systems that may improve its competitiveness of iron to benefit the fitness on host plants.

Though the pathogenesis mechanism of *Enterobacter* remains to be fully elucidated, our data may provide an insight into potential processes related to bacterial virulence. In the future, it is necessary to pay efforts on linking bacterial virulence and underlying genetic determinants, which can pave the way for understanding of serious disease development by novel pathogens.

## Supplementary Information

**Additional file 1: Figure S1. **The CRISPR region prediction by CRISPRCasFinder.* E.mori *CX01 contains two putative CRISPR repeat consensus sequences at evidence level4.

**Additional file 2: Table S1A.** Accession numbers of gene sequences used in the MLSA analaysis. **Table S1B.** Accession numbers of genomes used in the MLSA analaysis. **Table S2. **A set of 111 COGs specifically belongs to *E. mori CX01 and LMG 25706*. **Table S3.** Specific Cluster of Orthologous Groups in *Enterobacter mori CX01*. **Table S4.** Specific Cluster of Orthologous Groups in *Enterobacter mori LMG 25706*. **Table S5.** Comparison of carbohydrate metabolism pathway in *E. mori CX01 and LMG 25706 and Psa*. **Table S6. **Subsystem Feature Category counts in *Enterobacter mori* strains. **Table S7. **Flagellar genes. **Table S8. **Two-component systems of histidine kinase in *E. mori CX01*. **Table S9. **ABC transporters in *E. mori CX01*. **Table S10. **PTS systems in *E. mori CX01*. **Table S11. **MFS transporters in *E. mori CX01*. **Table S12. **Carbohydrate metabolism. **Table S13.** Phosphotransferase system (PTS) [PATH:ko02060]. **Table S14. **Biofilm formation—Escherichia coli [PATH:ko02026]. **Table S15. **Peptidases and inhibitors [BR:ko01002]. **Table S16. **Transporters [BR:ko02000]. **Table S17. **Transcription factors [BR:ko03000].

**Additional file 3. **Genome annotation of *Enterobacter mori* CX01.

**Additional file 4.** Nucleic acid and protein sequences derived from *Enterobacter mori* CX01 open reading frames.

## Data Availability

All data generated or analyzed are included in this article and its additional files. For further information, please contact the author.
