# Supplementary material for: Whole genome sequencing of Enterobacter mori, an emerging pathogen of kiwifruit and the potential genetic adaptation to pathogenic lifestyle
Source: AMB Express. 2021 Sep 17;11:129. doi: 10.1186/s13568-021-01290-w (PMC8448808; doi:10.1186/s13568-021-01290-w)
Supplement: Supplementary file 1 — Additional file 1: Figure S1. The CRISPR region prediction by CRISPRCasFinder. E.mori CX01 contains two putative CRISPR repeat consensus sequences at evidence level4. [file 13568_2021_1290_MOESM1_ESM.pdf]

AMB Express

Whole genome sequencing of *Enterobacter mori*, an emerging pathogen of kiwifruit and the potential genetic adaptation to pathogenic lifestyle

Mingyang Zhang[1,2,#], Yilin Zhang[3,#], Xue Han[3], Juan Wang[1,2], Yu Yang[3], Biao Ren[1,2,4], Mian Xia[5], Gang Li[5,6], Rongxiang Fang[1,2], Hang He[3,*], Yantao Jia[1,2,*]

[1]State Key Laboratory of Plant Genomics, Institute of Microbiology, Chinese Academy of Sciences, Beijing 100101, China

[2]National Plant Gene Research Center, Beijing 100101, China

[3] School of Advanced Agriculture Sciences and School of Life Sciences, State Key Laboratory of Protein and Plant Gene Research, Peking University, Beijing 100871, China

[4]College of Life Sciences, University of the Chinese Academy of Sciences, Beijing 100049, China [4]

[5] Cangxi Xingke Modern Agricultural Science and Technology Research Institute Co., Ltd., Cangxi, 628400, China

[6]Guangxi Key Laboratory of Medicinal Resource Protection and Genetic Improvement, Guangxi Botanical Garden of Medicinal Plant, Nanning, 530023, China

#Co-first authors

*Corresponding authors

hehang@pku.edu.cn Tel and Fax: 8610-62764981; jiayt@im.ac.cn Tel: 8610-64806884

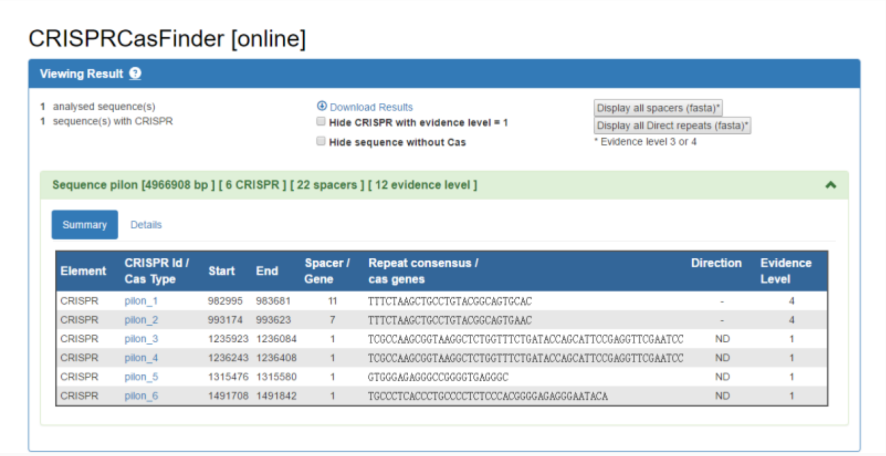
CRISPRCasFinder [online]

Viewing Result

1 analysed sequence(s)
1 sequence(s) with CRISPR

Download Results
Hide CRISPR with evidence level = 1
Hide sequence without Cas

Display all spacers (fasta)*
Display all Direct repeats (fasta)*
* Evidence level 3 or 4

Sequence pilon [4966908 bp] [6 CRISPR] [22 spacers] [12 evidence level]

Summary Details

Element	CRISPR Id / Cas Type	Start	End	Spacer / Gene	Repeat consensus / cas genes	Direction	Evidence Level
CRISPR	pilon_1	982995	983681	11	TTTCTAAGCTGCCTGTACGGCAGTGCAC	-	4
CRISPR	pilon_2	993174	993623	7	TTTCTAAGCTGCCTGTACGGCAGTGAAC	-	4
CRISPR	pilon_3	1235923	1236084	1	TCGCCAAGCGGTAAGGCTCTGGTTTCTGATACCAGCATTCCGAGGTTCGAATCC	ND	1
CRISPR	pilon_4	1236243	1236408	1	TCGCCAAGCGGTAAGGCTCTGGTTTCTGATACCAGCATTCCGAGGTTCGAATCC	ND	1
CRISPR	pilon_5	1315476	1315580	1	GTGGGAGAGGGCCGGGGTGAGGGC	ND	1
CRISPR	pilon_6	1491708	1491842	1	TGCCCTCACCCTGCCCCTCTCCCACGGGGAGAGGGAATACA	ND	1

Figure S1. The CRISPR region prediction by CRISPRCasFinder. *E. mori* CX01 contains two putative CRISPR repeat consensus sequences at evidence level 4.
